# Efficacy and safety of sofosbuvir–velpatasvir with or without ribavirin in HCV-infected Japanese patients with decompensated cirrhosis: an open-label phase 3 trial

**DOI:** 10.1007/s00535-018-1503-x

**Published:** 2018-09-10

**Authors:** Tetsuo Takehara, Naoya Sakamoto, Shuhei Nishiguchi, Fusao Ikeda, Tomohide Tatsumi, Yoshiyuki Ueno, Hiroshi Yatsuhashi, Yasuhiro Takikawa, Tatsuo Kanda, Minoru Sakamoto, Akihiro Tamori, Eiji Mita, Kazuaki Chayama, Gulan Zhang, Shampa De-Oertel, Hadas Dvory-Sobol, Takuma Matsuda, Luisa M. Stamm, Diana M. Brainard, Yasuhito Tanaka, Masayuki Kurosaki

**Affiliations:** 1 Osaka University, 2-15 Yamadaoka, Suita, Osaka, 565-0871 Japan; 2 Hokkaido University, Sapporo, Hokkaido Japan; 3 Hyogo College of Medicine, Nishinomiya, Hyogo Japan; 4 Okayama University, Okayama, Japan; 5 Yamagata University, Yamagata, Japan; 6 Nagasaki Medical Center, Nagasaki, Japan; 7 Iwate Medical University, Iwate, Japan; 8 Chiba University, Chiba, Japan; 9 University of Yamanashi, Yamanashi, Japan; 10 Osaka City University, Osaka, Japan; 11 National Hospital Organization Osaka National Hospital, Osaka, Japan; 12 Hiroshima University, Hiroshima, Japan; 13 Gilead Sciences, Inc, Foster City, CA USA; 14 Gilead Sciences K.K, Tokyo, Japan; 15 Nagoya City University, Nagoya, Aichi Japan; 16 Musashino Red Cross Hospital, Tokyo, Japan

**Keywords:** Sofosbuvir, Velpatasvir, Decompensated cirrhosis, Advanced liver disease, Direct-acting antivirals

## Abstract

**Background:**

In Japan, hepatitis C virus (HCV)-infected patients with decompensated cirrhosis currently have no treatment options. In this Phase 3 study, we evaluated sofosbuvir–velpatasvir with or without ribavirin for 12 weeks in patients with any HCV genotype and decompensated cirrhosis [Child–Pugh–Turcotte (CPT) class B or C] in Japan.

**Methods:**

Patients were randomized 1:1 to receive sofosbuvir–velpatasvir with or without ribavirin for 12 weeks. Randomization was stratified by CPT class and genotype. Sustained virologic response 12 weeks following completion of treatment (SVR12) was the primary efficacy endpoint.

**Results:**

Of the 102 patients enrolled, 57% were treatment naive, 78% and 20% had genotype 1 and 2 HCV infection, respectively, and 77% and 20% had CPT class B and C cirrhosis, respectively, at baseline. Overall, 61% of patients were female and the mean age was 66 years (range 41–83). SVR12 rates were 92% (47/51) in each group. Among patients who achieved SVR12, 26% had improved CPT class from baseline to posttreatment week 12. Most adverse events (AEs) were consistent with clinical sequelae of advanced liver disease or known toxicities of ribavirin. Four patients (8%) who received sofosbuvir–velpatasvir and seven (14%) who received sofosbuvir–velpatasvir plus ribavirin experienced a serious AE. The 3 deaths (bacterial sepsis, gastric varices hemorrhage, hepatocellular carcinoma) were attributed to liver disease progression.

**Conclusion:**

Sofosbuvir–velpatasvir for 12 weeks provides a highly effective and well-tolerated therapy for Japanese patients with HCV and decompensated cirrhosis. Ribavirin did not improve efficacy but increased toxicity.

**Electronic supplementary material:**

The online version of this article (10.1007/s00535-018-1503-x) contains supplementary material, which is available to authorized users.

## Introduction

Globally, the treatment of HCV infection has been transformed with the development of direct-acting antiviral (DAA) agents, which target viral proteins and cellular processes essential to viral replication. These interferon-free, DAA-based regimens are generally well-tolerated and result in high rates of sustained virologic response (SVR) across most patient populations. However, some regimens containing protease inhibitors have been associated with hepatotoxicity and hepatic decompensation, particularly in patients with advanced cirrhosis thus precluding their use in some patients, including those with decompensated cirrhosis [1]. In contrast, ledipasvir/sofosbuvir and sofosbuvir/velpatasvir have demonstrated both safety and efficacy in patients with decompensated liver disease [2–4]. These studies were conducted in North America, Europe, Australia, and New Zealand. Data are lacking in Japanese patients, and there are no approved antiviral therapies currently available for this population in Japan. The current Japan Society of Hepatology (JSH) guidelines therefore do not recommend the use of DAA agents in patients with decompensated cirrhosis due to lack of safety or efficacy data in Japanese patients [5].

Of the approximately 1.0–1.5 million people chronically infected with hepatitis C virus (HCV) in Japan [5], approximately 35,000–50,000 may have decompensated cirrhosis [6, 7]. Liver transplantation is a potential nonpharmaceutical intervention; however, it is not commonly done in Japan, with only 438 liver transplants performed in 2016 [8]. Patients with decompensated cirrhosis are at high risk for development of hepatocellular carcinoma (HCC), bleeding diatheses, and fulminant infections. One-year survival rates in patients with Child–Pugh–Turcotte (CPT) class B or CPT class C cirrhosis are 80% and 45%, respectively [9, 10]. A retrospective cohort study of Japanese patients with CPT class C cirrhosis on the liver transplant registry demonstrated a mean survival time of less than 16 months and 2-year survival probability was less than 40% [11]. Without available antiviral therapy and limited options for liver transplantation in Japan [8], the prognosis for Japanese patients with chronic HCV infection and decompensated cirrhosis is poor. A safe and effective HCV treatment will address the unmet medical need for this population.

Sofosbuvir–velpatasvir (400/100 mg) is a fixed-dose combination that combines 2 DAAs. Sofosbuvir is a nucleotide analog that is a potent, pangenotypic and selective NS5B polymerase inhibitor, and velpatasvir is a potent, pangenotypic, next-generation HCV NS5A inhibitor. Sofosbuvir–velpatasvir is approved in the US, European Union, and other regions for the treatment of genotypes 1–6 chronic HCV infection in patients with and without compensated cirrhosis and for use with ribavirin in patients with decompensated cirrhosis [12, 13].

The ASTRAL-4 study evaluated 12 and 24 weeks of treatment with sofosbuvir–velpatasvir with or without ribavirin in HCV-infected patients with CPT class B decompensated cirrhosis in the US [4]. Rates of sustained virologic response 12 weeks post treatment (SVR12) were 83% in patients who received 12 weeks of sofosbuvir–velpatasvir, 94% in patients who received 12 weeks of sofosbuvir–velpatasvir plus ribavirin, and 86% in patients who received 24 weeks of sofosbuvir–velpatasvir. Notably, the numeric difference in SVR12 rates in genotype 1b and genotype 2 HCV-infected patients who received sofosbuvir–velpatasvir for 12 weeks or sofosbuvir–velpatasvir with ribavirin for 12 weeks did not differ substantially.

In this Phase 3 study, we evaluated the efficacy and safety of the fixed-dose combination tablet of sofosbuvir–velpatasvir with or without ribavirin for 12 weeks in Japanese HCV-infected patients with decompensated cirrhosis.

## Methods

### Patients

Eligible patients were 20 years of age and older with chronic HCV infection, quantifiable HCV RNA at screening, and CPT score 7–12, inclusive. The calculation of the CPT score at screening used either the international normalized ratio or prothrombin activation percentage for the coagulation parameter, at the investigator’s discretion (Supplemental Table 1). Patients were to have liver imaging within 4 months of baseline to exclude HCC. Patients were excluded from this study if they had a positive test result for hepatitis B surface antigen or human immunodeficiency virus, had HCC within 2 years prior to screening, any recurrence of HCC after curative treatment (e.g., successful treatment with surgical resection or radiofrequency ablation), prior treatment with an NS5A inhibitor, or creatinine clearance < 50 mL/min as calculated by the Cockcroft–Gault equation using actual body weight. Use of concomitant amiodarone was prohibited from 60 days prior to day 1 and throughout the treatment period. Full eligibility criteria are provided in the supplementary information.

### Study design and randomization

This was a Phase 3, multicenter, open-label study. Via an interactive web response system, patients were randomly assigned 1:1 to sofosbuvir–velpatasvir with or without ribavirin for 12 weeks. Randomization was stratified by genotype (genotype 1 vs. non-genotype 1) and CPT class at screening (CPT class B vs C). For the purposes of randomization, a patient with nondefinitive or mixed HCV genotype results was considered non-genotype 1. Across the study population, at least 15 patients were to have non-genotype 1 HCV infection and approximately 10% of patients were to have CPT class C cirrhosis. Enrollment of patients with CPT class C cirrhosis began after an independent data monitoring committee evaluated the safety data through 4 weeks of treatment from the first 20 patients with CPT class B cirrhosis.

Sofosbuvir–velpatasvir (400/100 mg) fixed-dose combination was administered once daily. Ribavirin (REBETOL, MSD KK) was administered with food twice daily. For patients with CPT class B cirrhosis at screening dosing was based on body weight (600 mg daily in patients ≤ 60 kg, 800 mg for patients > 60–80 kg, and 1000 mg for those > 80 kg). All patients with CPT class C cirrhosis received 600 mg daily regardless of weight.

All patients provided written informed consent to participate, and the study was conducted consistent with the ethical standards, including but not limited to the International Council for Harmonisation guideline for Good Clinical Practice, the original principles embodied in the Declaration of Helsinki, and the J-GCP (Ministerial Ordinance on Good Clinical Practice for Drugs). This study was approved by an institutional review board at each study site prior to the initiation of any screening or study-specific procedures.

### Study assessments

Screening assessments included HCV genotyping, *IL28B* genotyping, and standard laboratory and clinical tests. HCV genotype and subtype were determined using the Siemens VERSANT HCV Genotype INNO-LiPA2.0 Assay. *IL28B* genotype was determined by polymerase chain reaction amplification of the single-nucleotide polymorphism rs12979860, with sequence-specific forward and reverse primers and allele-specific fluorescently labeled TaqMan minor groove binder probes. Plasma HCV RNA levels were evaluated at screening; at day 1 of treatment, at weeks 2, 4, 8, and 12 during treatment, and at weeks 4, 12, and 24 after the end of treatment. HCV RNA levels were quantified using the COBAS Ampliprep/COBAS TaqMan HCV Test, v2.0 (Roche Molecular Systems, Inc., Branchburg, NJ), which has a lower limit of quantification (LLOQ) of 15 IU/mL.

Deep sequencing of the HCV NS5A and NS5B genes was performed for all patients at baseline and from those with virologic failure at the time of failure (DDL Diagnostic Laboratory, Rijswijk, Netherlands). RASs present in more than 15% of the sequence reads are reported. The resistance analysis population is comprised of patients with viral sequence data and virologic outcome data available.

Safety assessments included monitoring of adverse events (AEs) and clinical laboratory tests at all on-treatment visits; AEs were also collected up to 30 days after the last dose of study drug. Samples for clinical laboratory tests were collected at each posttreatment visit (4, 12, and 24 weeks after the last dose of study drug). All AEs and laboratory values were graded according to a standardized scale and AEs were coded using the Medical Dictionary for Regulatory Activities (MedDRA), Version 20.1.

### Endpoints

The primary efficacy endpoint was SVR12, defined as HCV RNA < LLOQ (i.e., < 15 IU/mL) 12 weeks after the end of treatment. Secondary efficacy endpoints included the change from baseline in the CPT and MELD scores at 12 weeks after end of treatment. CPT score for all baseline and post-baseline visits were calculated using prothrombin activation percentage for the coagulation parameter. The primary safety endpoint was discontinuation of study drugs due to AEs.

### Statistical analysis

Point estimates with 2-sided 95% exact confidence intervals (CIs) for SVR12 based on the Clopper–Pearson method were provided for each treatment group. In the primary efficacy analysis, the SVR12 rate for patients in each treatment group was compared to the spontaneous clearance rate of 1% using a 2-sided exact 1-sample binomial test with Bonferroni alpha adjustment (each at the 0.025 significance level).

## Results

### Baseline characteristics and disposition

Demographics and baseline characteristics are presented in Table 1. Of 155 patients screened, a total of 102 patients were enrolled at 33 sites in Japan, of which 100 (98%) completed treatment (Supplemental Fig. 1). All 53 patients who were excluded from study participation did not meet eligibility criteria (Supplemental Table 2). Demographics and baseline characteristics of the patients enrolled were generally balanced across both treatment groups and consistent with an older population with advanced liver disease. Overall, most patients were female (61%). The mean age was 66 years (range 41–83), and 58% were ≥ 65 years of age. Most patients had *IL28B* CC genotype (69%) and were treatment naive (57%). Among the 44 treatment-experienced patients, only 1 had previously been treated with a DAA (simeprevir in combination with peginterferon alfa-2a and ribavirin for 23 weeks); all others had been treated with interferon alone or in combination with ribavirin.

**Table 1:** Baseline demographics and disease characteristics

	Sofosbuvir–velpatasvir 12 weeks *N* = 51	Sofosbuvir–velpatasvir plus ribavirin 12 weeks *N* = 51
Mean age (range) (years)	66 (43, 82)	66 (41, 83)
Female sex	33 (65)	29 (57)
Mean body mass index (range) (kg/m^2^)	26.5 (20.4, 43.0)	25.8 (18.3, 58.6)
HCV genotype and subtype		
Genotype 1	41 (80)	39 (76)
Genotype 1a	1 (2)	0
Genotype 1b	40 (78)	39 (76)
Genotype 2	9 (18)	11 (22)
Genotype 2 (no confirmed subtype)	5 (10)	5 (10)
Genotype 2a	0	2 (4)^a^
Genotype 2a/2c	2 (4)	1 (2)
Genotype 2b	2 (4)	4 (8)
Genotype 3b	1 (2)	0
Mean HCV RNA (range) (log_10_ IU/mL)	5.7 (3.7–7.1)	5.8 (4.2–7.0)
IL28B CC genotype	33 (65)	37 (73)
CPT B [7–9]^b^	40 (78)	39 (76)
MELD score ≤ 15	46 (90)	48 (94)
Ascites		
None	19 (37)	16 (31)
Mild/moderate	32 (63)	33 (65)
Severe	0	2 (4)
Encephalopathy		
None	23 (45)	22 (43)
Medication-controlled	28 (55)	29 (57)
No prior HCV treatment	27 (53)	31 (61)
Mean estimated glomerular filtration rate (range) (mL/min)^c^	93 (40, 183)	89 (42, 299)

Data presented are *n* (%) unless stated otherwise

*CPT* Child–Pugh–Turcotte

^a^One patient with missing HCV genotype was subsequently determined to have genotype 2a HCV infection by BLAST analysis

^b^The CPT score was calculated using prothrombin activation percentage for the coagulation parameter

^c^The estimated glomerular filtration rate was calculated using the Cockcroft–Gault equation

Overall, 80 patients (78%) had genotype 1 HCV infection [1 patient (1%) had HCV genotype 1a and 79 (77%) patients had HCV genotype 1b], 20 patients (20%) had genotype 2 HCV infection, and 1 patient (1%) had genotype 3 HCV infection. There was 1 patient who had an HCV genotype that was unable to be determined by LiPA or NS5B Sanger, but was later determined to have genotype 2a HCV infection by BLAST analysis. At baseline, 77% of patients were CPT class B (score 7–9), 20% were CPT class C (score 10–12), and 3% were CPT class A (score 6).

## Efficacy

### Virologic response

The SVR12 rates were 92% (47/51; 95% CI 81–98%) in each treatment group (Table 2). Both treatment groups met their primary efficacy endpoints with SVR12 rates that were statistically superior compared with the spontaneous clearance rate of 1% (*p* < 0.001).

**Table 2:** Virologic response during and after treatment

	Sofosbuvir–velpatasvir 12 weeks *N* = 51	Sofosbuvir–velpatasvir plus ribavirin 12 weeks *N* = 51
HCV RNA < 15 IU/mL, *n*/*n* (%)		
On treatment		
Week 2	23/51 (45)	26/51 (51)
Week 4	49/51 (96)	46/51 (90)
Week 8	51/51 (100)	49/51 (96)
Week 12	51/51 (100)	49/49 (100)
After treatment		
Week 4 (SVR4)	48/51 (94)	49/51 (96)
Week 12 (SVR12)	47/51 (92)	47/51 (92)
95% CI	81–98	81–98
Relapse after the end of treatment	4 (8)	2 (4)
Discontinued treatment due to adverse events	0	2 (4)

When examined by genotype, SVR12 rates were high for patients with genotype 1 or 2 regardless if they received 12 weeks of sofosbuvir–velpatasvir or sofosbuvir–velpatasvir plus ribavirin (rates ranged from 89 to 100%, Table 3). The 1 patient with genotype 3 HCV infection in the study who was randomized to the sofosbuvir–velpatasvir group did not achieve SVR12. When examined by baseline CPT class, SVR12 rates were high in patients with CPT class B cirrhosis (≥ 95%) in both treatment groups (Table 3). Of the patients with baseline CPT class C cirrhosis, 80% (8/10) and 70% (7/10) in the sofosbuvir–velpatasvir and sofosbuvir–velpatasvir plus ribavirin groups, respectively, achieved SVR12.

**Table 3:** Rates of SVR12 by subgroup

	Sofosbuvir–velpatasvir 12 weeks *N* = 51	Sofosbuvir–velpatasvir plus ribavirin 12 weeks *N* = 51
Overall SVR12	47/51 (92)	47/51 (92)
Genotype		
1a	0/1 (0)	–
1b	39/40 (98)	35/39 (90)
2	8/9 (89)	12/12 (100)^a^
3	0/1 (0)	–
Baseline CPT class		
A	1/1 (100)	2/2 (100)
B	38/40 (95)	38/39 (97)
C	8/10 (80)	7/10 (70)

^a^Includes 1 patient who was initially categorized as missing HCV genotype, and subsequently determined to have genotype 2a by BLAST analysis

A total of 8 patients did not achieve SVR12, with 6 patients experiencing virologic relapse (Supplemental Table 3). No patients had virologic non-response. In the sofosbuvir–velpatasvir group, 4 of 51 patients (8%) relapsed. In the sofosbuvir–velpatasvir plus ribavirin group, 4 of 51 patients (8%) did not achieve SVR12. Of these 4 patients, 2 relapsed and 2 discontinued treatment early due to AEs and subsequently died.

### Changes in liver function

Of all patients who achieved SVR12 in either arm, 26% (24/91) improved in CPT class and 2% (2/91) worsened in CPT class from baseline to posttreatment week 12 (Table 4). Improvement in CPT score was primarily driven by increase in albumin levels with 79% of the patients with improved CPT scores having increase in albumin (Supplemental Table 4). Similar changes were observed in MELD score with 27% (25/94) having improved MELD score and 15% (14/94) with worsening MELD score.

**Table 4:** Shift of CPT class from baseline to posttreatment week 12

Posttreatment week 12 CPT class, *n* (%)	Overall *N* = 94		
	Baseline CPT class		
	CPT A (5–6) *N* = 3	CPT B (7–9) *N* = 76	CPT C (10–15) *N* = 15
CPT A (5–6)	3 (100)	19 (25)	0
CPT B (7–9)	0	55 (72)	5 (33)
CPT C (10–15)	0	2 (3)	10 (67)

*CPT* Child–Pugh Turcotte

### Analysis of resistance

Among the 100 patients included in the resistance analysis population, 41% (41/100) had baseline NS5A RASs. No patient had NS5B nucleoside inhibitor (NI) RASs.

In the sofosbuvir–velpatasvir group, 97% (33/34) of patients without baseline NS5A RASs and 82% (14/17) of patients with baseline NS5A RASs achieved SVR12. Of the 41 patients with genotype 1 HCV infection, there was 1 patient without baseline NS5A RASs and 1 patient with baseline NS5A RASs who relapsed. In the sofosbuvir–velpatasvir plus ribavirin group, 96% (24/25) of patients without baseline NS5A RASs and 96% (23/24) of patients with baseline NS5A RASs achieved SVR12. Of the 37 patients with genotype 1 HCV infection, there was 1 patient without baseline NS5A RASs and 1 patient with baseline NS5A RASs who relapsed.

Of the 6 patients who experienced virologic relapse across both treatment groups, 4 had treatment-emergent NS5A RASs. No patient in either treatment group had NS5B NI RASs detected at baseline or relapse.

### Safety

More patients treated with sofosbuvir–velpatasvir plus ribavirin experienced AEs (86%, 44/51) compared with patients treated with sofosbuvir–velpatasvir (69%, 35/51) (Table 5). No consistent, clinically significant trends were observed when looking at AE rates by CPT class, nor by age group.

**Table 5:** Adverse events and grade 3 and 4 laboratory abnormalities

	Sofosbuvir–velpatasvir 12 weeks *N* = 51	Sofosbuvir–velpatasvir plus ribavirin 12 weeks *N* = 51
Number (%) of patients experiencing any		
Adverse event	35 (69)	44 (86)
Grade 3 or above adverse event	2 (4)	5 (10)
Serious adverse event	4 (8)	7 (14)
Adverse event leading to discontinuation of sofosbuvir/velpatasvir	0	2 (4)
Adverse event leading to discontinuation of ribavirin	N/A	9 (18)
Adverse event leading to modification or interruption of ribavirin	N/A	18 (35)
Deaths	0	3 (6)
Common adverse events (≥ 10% either group)		
Anemia	0	20 (39)
Nasopharyngitis	7 (14)	3 (6)
Diarrhea	0	7 (14)
Laboratory abnormalities (≥ 10% either group)		
Hemoglobin < 10 g/dL	2 (4)	7 (14)
Lymphocytes, < 500/mm^3^	0	5 (10)
Platelets, 25,000–50,000/mm^3^	1 (2)	6 (12)
Hyperglycemia, > 250–500 mg/dL	5 (10)	9 (18)
Total bilirubin, > 2.5 × ULN	6 (12)	12 (24)

Toxicity grade must have increased at least 1 toxicity grade from baseline value (missing was considered grade 0) to be included. Patients were counted once at maximum toxicity grade for each laboratory test. Data were included up to the last dose date of any study drug + 30 days

Despite all the patients in the study having advanced liver disease, most AEs reported in this study were Grade 1 (mild) or Grade 2 (moderate) in severity. The most common AEs in the sofosbuvir–velpatasvir group were nasopharyngitis (14%) and in the sofosbuvir–velpatasvir plus ribavirin group they were anemia (39%) and diarrhea (14%).

Patients in the sofosbuvir–velpatasvir plus ribavirin group experienced AEs consistent with ribavirin toxicity. Eighteen of 51 patients (35%) had AEs that led to modification or interruption of ribavirin and 9 patients (18%) had AEs that led to discontinuation of ribavirin, with anemia being the most common in both instances.

Four patients (8%) in the sofosbuvir–velpatasvir group and 7 patients (14%) in the sofosbuvir–velpatasvir plus ribavirin group had serious adverse events (SAEs), and most were not considered treatment-related by the investigator (Supplemental Table 5). The only SAEs that occurred in > 1 patient were femur fracture (2 in the sofosbuvir–velpatasvir plus ribavirin group) and hepatic encephalopathy (1 in the sofosbuvir–velpatasvir group, 2 in the sofosbuvir–velpatasvir plus ribavirin group). Two of the three SAEs of hepatic encephalopathy occurred in patients with CPT class C cirrhosis.

Three patients in the study developed HCC, all of whom were diagnosed following treatment (on posttreatment day 1, posttreatment day 70 and posttreatment day 124). Two of the patients had CPT class B at baseline and one had CPT class C. The investigator did not consider these events related to study drug. There were 4 patients enrolled who had a history of HCC, none of whom experienced recurrence during the study.

Three deaths occurred during the study and all 3 patients received treatment with sofosbuvir–velpatasvir plus ribavirin. The ages of the patients who died were 51, 59 and 67 years; all 3 patients had CPT class C at baseline. Two of these patients discontinued study drugs early due to AEs not related to treatment. All 3 deaths occurred after treatment was stopped (posttreatment days 5 and 17 for the 2 patients that discontinued study drugs prematurely, and posttreatment day 158 for the patient that completed 12 weeks of study treatment). All of the deaths were due to progression of end-stage liver disease (septicemia, portal hypertension leading to gastrointestinal bleeding, and HCC) and none were considered to be related to study drugs by the investigator (Supplemental Table 6). No other patients discontinued sofosbuvir–velpatasvir in the study.

Fewer patients in the sofosbuvir–velpatasvir group had Grade 3 or 4 laboratory abnormalities compared with the sofosbuvir–velpatasvir plus ribavirin group (27 vs 53%, respectively) (Table 5). The observed laboratory abnormalities were consistent with those expected in a population with decompensated liver disease and, in the sofosbuvir–velpatasvir plus ribavirin group, consistent with the known toxicities of ribavirin. Post-baseline hemoglobin values < 10 g/dL were observed in 2 patients (4%) in the sofosbuvir–velpatasvir group and 7 patients (14%) in the sofosbuvir–velpatasvir plus ribavirin group. Additional information about laboratory abnormalities is provided in the supplementary information (Supplemental Fig. 2).

## Discussion

In this Phase 3 study conducted in Japan, sofosbuvir–velpatasvir for 12 weeks was highly effective and generally safe and well-tolerated in patients with decompensated cirrhosis. The current study enrolled mostly patients with genotype 1b or 2, consistent with the Japanese population of HCV-infected patients. The identical SVR12 rates of 92% in the 2 treatment groups suggest that addition of ribavirin to sofosbuvir–velpatasvir did not improve efficacy for Japanese patients with decompensated cirrhosis. These results were comparable to those for the similar subpopulation enrolled in the ASTRAL-4 study, in which 12 weeks of treatment with sofosbuvir–velpatasvir without ribavirin resulted in SVR12 rates of 89% (16 of 18) and 100% (4 of 4) in patients with genotype 1b and 2, respectively [4]. Of note, the addition of ribavirin was most beneficial in patients with genotype 3 HCV infection in the ASTRAL-4 study, where the response was 35% higher in the group who received ribavirin (85%, 11 of 13 patients) compared to those who did not in either the sofosbuvir–velpatasvir 12 week group (50%, 7 of 14 patients) or 24 week group (50%, 6 of 12 patients).

Clinical attention to safety is appropriate in this patient population with advanced liver disease with high expected morbidity and mortality. In the current study, the AE profile was consistent with the clinical sequelae of advanced liver disease and with the known toxicities of ribavirin. In the sofosbuvir–velpatasvir plus ribavirin group, 49% of patients needed significant modifications to their ribavirin dosing, primarily due to anemia. Overall sofosbuvir–velpatasvir was well-tolerated with the majority of AEs being Grade 1 or 2. Only 2 patients, both in the sofosbuvir–velpatasvir plus ribavirin group, discontinued sofosbuvir–velpatasvir for AEs that were not considered related to study drugs; both of these patients subsequently died due to progression of their liver disease. The safety profile observed in the current study, including the rate of deaths, was consistent with those observed in previous overseas trials of sofosbuvir–velpatasvir with and without ribavirin as well as ledipasvir–sofosbuvir with ribavirin in larger populations of patients with decompensated cirrhosis, despite the fact that the mean age of patients in the current study was 8–9 years older than in the overseas studies [2–4].

As interferon-free DAA-based regimens have only recently become available for the treatment of HCV, the clinical benefits of their use in patients with decompensated cirrhosis are being characterized. Achievement of SVR12 is associated with early improvements in liver function, as demonstrated by reductions in CPT and MELD scores through posttreatment week 12, in both the current study as well as previous studies of sofosbuvir-based regimens in this population [2–4]. In terms of long-term benefits of achieving SVR with DAA-based regimens in patients with decompensated cirrhosis, several studies have compared the survival rates of patients successfully treated with sofosbuvir-based regimens to historical matched controls from transplant waitlists and have demonstrated a decrease in mortality [14, 15]. There is also a growing body of literature demonstrating a reduction in risk of de novo HCC, consistent with observations in the interferon era [16–18].

Our study has several limitations, mostly related to characteristics of the enrolled patients. Although representative of the Japanese HCV-infected patient population, there was a lack of genotype diversity. The study included few patients with more severe cirrhosis (CPT class C) and none with baseline CPT score greater than 12. Patients who had been previously treated with DAAs were not included. Lastly, although early improvements in liver function were demonstrated through the study posttreatment period, the long-term clinical benefit of achievement of SVR in patients with decompensated liver disease can only be demonstrated through follow-up of the patients after the study.

In conclusion, treatment with sofosbuvir–velpatasvir for 12 weeks is the optimal regimen for Japanese patients with decompensated cirrhosis. The SVR12 rate was high regardless of genotype or CPT class. Addition of ribavirin to the regimen did not improve efficacy and was associated with more adverse events and laboratory abnormalities.

## Electronic supplementary material

Below is the link to the electronic supplementary material.

Supplementary material 1 (DOCX 572 kb)

## Abbreviations

AE: Adverse event
BMI: Body mass index
CI: Confidence interval
CPT: Child–Pugh–Turcotte
DAA: Direct-acting antiviral
HCC: Hepatocellular carcinoma
HCV: Hepatitis C virus
LLOQ: Lower limit of quantification
MELD: Model for end-stage liver disease
NI: Nucleoside inhibitor
RAS: Resistance-associated substitution
RNA: Ribonucleic acid
SAE: Serious adverse event
SVRxx: Sustained virologic response at “xx” weeks following completion of treatment
