# Supplementary material for: Efficacy and safety of sofosbuvir–velpatasvir with or without ribavirin in HCV-infected Japanese patients with decompensated cirrhosis: an open-label phase 3 trial
Source: J Gastroenterol. 2018 Sep 10;54(1):87–95. doi: 10.1007/s00535-018-1503-x (PMC6314981; doi:10.1007/s00535-018-1503-x)

Supplementary appendix to:

# Efficacy and safety of sofosbuvir-velpatasvir with or without ribavirin in HCV-infected Japanese patients with decompensated cirrhosis: an open-label phase 3 trial

**Table of Contents**

GS-US-342-4019 Study Investigators 3

Supplemental Table 1. Child-Pugh-Turcotte Scoring of the Severity of Cirrhosis 4

Full Eligibility Criteria 5

Supplemental Figure 1. Patient Disposition 10

Supplemental Table 2. Reasons for screen failure 11

Supplemental Table 3. Characteristics of patients with virologic relapse 12

Supplemental Table 4. Components of CPT-PT% for Patients with Improvement in CPT Score 13

Supplemental Table 5, Serious Adverse Events 14

Supplemental Table 6. Deaths 15

Supplemental Figure 2. Selected laboratory parameters during study treatment 16

**GS-US-342-4019 Study Investigators**

**Japan**

Yasuhiro Asahina, Hirayuki Enomoto, Shigetoshi Fujiyama, Takuya Genda,  Yoichi Hiasa, Tatsuya Ide, Fusao Ikeda,  Masatoshi Ishigami, Jun Inoue, Yoshito Itoh, Naoya Kato,  Kazuhiko Koike, Masayuki Kurosaki, Yasushi Matsuzaki, Eiji Mita, Masashi Mizokami, Satoshi Mochida, Kenta Motomura, Kei Moriya, Toshimitsu Murohisa, Takashi Nakahara, Kazuo Notsumata,  Koji Ogawa, Masao Omata, Kohsaku Sakaguchi, Minoru Sakamoto,  Koichi Takaguchi, Taro Takami,  Yasuhiro Takikawa, Akihiro Tamori, Yatsuhashi Tanaka, Tomohide Tatsumi, Yoshihide Ueda,  Yoshiyuki Ueno, Hirofumi Uto, Satoyoshi Yamashita, Hiroshi Yatsuhashi

**Supplemental Table 1. Child-Pugh-Turcotte Scoring of the Severity of Cirrhosis**

| **Measure** | | **1 point** | **2 points** | **3 points** |
| --- | --- | --- | --- | --- |
| Total bilirubin, mg/dL, (μmol/L) | | < 2, (< 34) | 2-3, (34-50) | > 3, (> 50) |
| Serum albumin, g/dL | | > 3.5 | 2.8-3.5 | < 2.8 |
| Coagulation^a^ | INR | < 1.7 | 1.7-2.3 | > 2.3 |
|  | Prothrombin  Activation  % | >70% | 40%-70% | < 40% |
| Ascites | | **None**  No ascites and not on treatment for ascites | **Mild/Moderate**  Cross sectional imaging  showing ascites  Abdominal distension  Medication for ascites | **Severe (diuretic-refractory)**  Visible clinically |
| Hepatic encephalopathy | | **None**  No encephalopathy and not on any treatment for hepatic encephalopathy | **Medication-Controlled**  Patient is lethargic, may have moderate confusion  Patient is receiving  medical therapy for hepatic encephalopathy | **Medication-Refractory**  Marked confusion/incoherent, rousable but sleeping unless aroused or comatosed |

a For coagulation, either INR or prothrombin activation % was used for screening at the investigator’s discretion. All analyses of CPT score at baseline and post-baseline used prothrombin activation % for the coagulation parameter.

**Full Eligibility Criteria**

**Inclusion criteria**

Patients who met all of the following inclusion criteria were eligible for participation in the study:

1. Willing and able to provide written informed consent
2. Male or female, aged ≥ 20 years at screening
3. Body weight ≥ 40 kg at screening
4. Quantifiable HCV RNA at screening
5. Chronic HCV infection (≥ 6 months prior to screening) documented by prior medical history or liver biopsy
6. Confirmation of cirrhosis by any one of the following methods:
   1. Liver biopsy showing cirrhosis (eg, Metavir score = 4 or Ishak score ≥ 5)
   2. Fibroscan^®^ showing cirrhosis as reflected by a result > 12.5 kPa
   3. In the absence of liver biopsy or availability of Fibroscan, a FibroTest^®^ score of  > 0.75 at screening
7. CPT score 7 to 12, inclusive, at screening as determined using laboratory values from the central laboratory, and at Day 1 (before dosing) using laboratory values from the local laboratory.
8. Liver imaging (eg, ultrasound or computed tomography scan, at the discretion of the investigator) performed within 4 months of Day 1 to exclude hepatocellular carcinoma.
9. If treatment-experienced, the most recent HCV treatment must have been completed at least 8 weeks prior to screening.
10. If listed for liver transplant, Day 1 was expected to be at least 12 weeks prior to transplant.
11. Females of childbearing potential must have had a negative serum pregnancy test at screening and a negative urine pregnancy test on Day 1 prior to randomization.
12. Male patients and female patients of childbearing potential who engaged in heterosexual intercourse must have agreed to use protocol‑specified method(s) of contraception.
13. Female patients must have agreed to refrain from egg donation and in vitro fertilization during treatment until at least 6 months after the last dose of ribavirin or 30 days after the last dose sofosbuvir/velpatasvir, whichever occurs last.
14. Male patients must have agreed to and refrained from sperm donation from the date of screening until at least 6 months after the last dose of ribavirin or 30 days after the last dose of sofosbuvir/velpatasvir, whichever occurs last.
15. Lactating females must have agreed to discontinue nursing before the study drugs were administered and through at least 12 weeks after the last dose of study drug.
16. Patient must have been able to comply with the dosing instructions for study drug administration and to complete the study schedule of assessments, including all required posttreatment visits.

**Exclusion criteria**

Patients with any of the following exclusion criteria were not eligible for participation in the study:

1. Current or prior history of any of the following:
   1. Clinically significant illness or currently under evaluation for a potentially clinically significant illness (other than HCV or co-morbidities associated with advanced liver disease except as noted below) or any other major medical disorder that may have interfered with patient treatment, assessment, or compliance with the protocol.
   2. Gastrointestinal disorder or postoperative condition that could have interfered with the absorption of the study drugs
   3. Difficulty with blood collection and/or poor venous access for the purposes of phlebotomy
   4. Solid organ transplantation
   5. Significant pulmonary disease
   6. Unstable cardiac disease or significant cardiac event within 1 year prior to screening
   7. Porphyria
   8. History of clinically significant hemoglobinopathy (eg, sickle cell disease, thalassemia)
   9. Psychiatric hospitalization, suicide attempt, and/or a period of disability as a result of their psychiatric illness within the last 2 years of screening
   10. Malignancy, other than hepatocellular carcinoma within the 5 years prior to screening, with the exception of specific cancers that are cured by surgical resection (eg, basal cell skin cancer). Patients under evaluation for possible malignancy were not eligible.
   11. Hepatocellular carcinoma within 2 years prior to screening. Patients with a history of HCC that was curatively treated with either surgical resection or radiofrequency ablation (RFA), with the end of treatment (EOT) occurring at least 2 years prior to screening, were eligible for enrollment.
   12. Recurrence of hepatocellular carcinoma after curative treatment
   13. Significant drug allergy (such as anaphylaxis or hepatotoxicity)
   14. Hepatopulmonary syndrome
   15. Hepatorenal syndrome
2. Infection with human immunodeficiency virus (HIV) at screening
3. Hepatitis B virus (HBV) surface antigen positive at screening
4. Screening electrocardiogram (ECG) with clinically significant abnormalities
5. Prior exposure to any HCV nonstructural protein (NS) 5A inhibitor
6. Use of granulocyte macrophage colony-stimulating factor (GM-CSF), epoetin alfa or other hematopoietic stimulating agents within 2 weeks of screening
7. Chronic liver disease of a non-HCV etiology (eg, hemochromatosis, autoimmune hepatitis, Wilson’s disease, α‑1‑antitrypsin deficiency, alcoholic liver disease, nonalcoholic steatohepatitis, or toxin exposures)
8. Pregnant or nursing female or male with a pregnant female partner
9. Women who wished to become pregnant or males with female partners who wished to become pregnant during study treatment and through 6 months after the last dose of ribavirin or 30 days after the last dose SOF/VEL whichever had come last
10. Chronic use of systemically administered immunosuppressive agents (eg, prednisone equivalent > 10 mg/day)
11. Active spontaneous bacterial peritonitis at screening
12. Infection requiring systemic antibiotics at the time of screening
13. Evidence of fibrosing cholestatic hepatitis
14. Life threatening serious adverse event (SAE) during screening
15. Active variceal bleeding within 6 months of screening
16. Prior placement of a portosystemic shunt (such as transjugular intrahepatic portosystemic shunt [TIPS])
17. Patients with any of the following laboratory parameters at screening:
    1. Hemoglobin < 11 g/dL for female patients; hemoglobin < 12 g/dL for male patients
    2. Platelets < 50,000/mm^3^
    3. Neutrophils < 1000/mm^3^
    4. Alanine aminotransferase (ALT), aspartate aminotransferase (AST), or alkaline phosphatase ≥ 10 x upper limit of normal (ULN)
    5. Sodium < 125 mEq/L
    6. Total bilirubin > 10 mg/dL
    7. Creatinine clearance (CL_cr_) < 50 mL/min as calculated by the Cockcroft‑Gault equation using actual body weight {[Cockcroft 1976](#_ENREF_10)}; ideal body weight could be used on a case‑by-case basis when approved by the medical monitor.
18. Donation or loss of more than 400 mL blood within 2 months prior to Day 1
19. Participation in a clinical study with an investigational drug or biologic within 1 month prior to screening
20. Any contraindication to ribavirin therapy, per the approved package insert, with the exception of serious hepatic dysfunction
21. Use of any prohibited concomitant medications as described in the below table:

| **Drug Class** | **Agents Disallowed** | **Use with Caution** |
| --- | --- | --- |
| Acid Reducing Agents^a^ |  | Proton- Pump Inhibitors, H2-Receptor Antagonists, Antacids |
| Anticonvulsants^b^ | Phenytoin, Carbamazepine, Phenobarbital, Oxcarbazepine |  |
| Antimycobacterials^b^ | Rifampicin, Rifabutin, Rifapentine^g^ |  |
| Cardiac Medications^c^ | Amiodarone^d^ | Digoxin^e^ |
| Herbal/Natural Supplements^b^ | St. John’s Wort, Echinacea, Milk thistle (ie, silymarin), Chinese herb sho-saiko-to (or Xiao-Shai-Hu-Tang) |  |
| HMG-CoA Reductase Inhibitors^f^ |  | Rosuvastatin (≤10 mg/day) ^f^, Atorvastatin |
| Other | Bosentan^b^, Modafinil ^b^, Sulfasalazine^c,^ , Methotrexate^c^ |  |

1. Proton pump inhibitor (PPI) doses comparable with omeprazole 20 mg can be administered with SOF/VEL when SOF/VEL is administered with food. H2‑receptor antagonists must not exceed a dose of 40 mg famotidine or equivalent and can be taken simultaneously with SOF/VEL and/or staggered by 12 hours. Antacids that directly neutralize stomach acid may not be taken within 4 hours (before or after) of SOF/VEL administration.
2. May result in a decrease in the concentration of study drugs.
3. May result in an increase in the concentration of study drugs and/or concomitant medications
4. May result in symptomatic bradycardia. Mechanism is not currently known. The use of amiodarone is prohibited from 60 days prior to Day 1 through the end of treatment
5. Monitor for signs and symptoms of digoxin toxicity.
6. Use with SOF/VEL may result in an increase in the concentration of HMG-CoA Reductase Inhibitor, rosuvastatin. Monitor for signs and symptoms of muscle weakness or myopathy, including rhabdomyolysis.
7. Unapproved in Japan
8. Known hypersensitivity to ribavirin, sofosbuvir, velpatasvir, or the metabolites or formulation excipients
9. Known contraindication to sofosbuvir per the approved package insert

**Supplemental Figure 1. Patient Disposition**

102 enrolled

53 did not meet eligibility criteria and were excluded

155 patients screened

2 discontinued treatment early due to adverse events

51 assessed for

efficacy and safety

51 assessed for

efficacy and safety

51 completed treatment

49 completed treatment

51 received

sofosbuvir-velpatasvir

for 12 weeks

51 received

sofosbuvir-velpatasvir+ribavirin

for 12 weeks

**Supplemental Table 2. Reasons for screen failure**


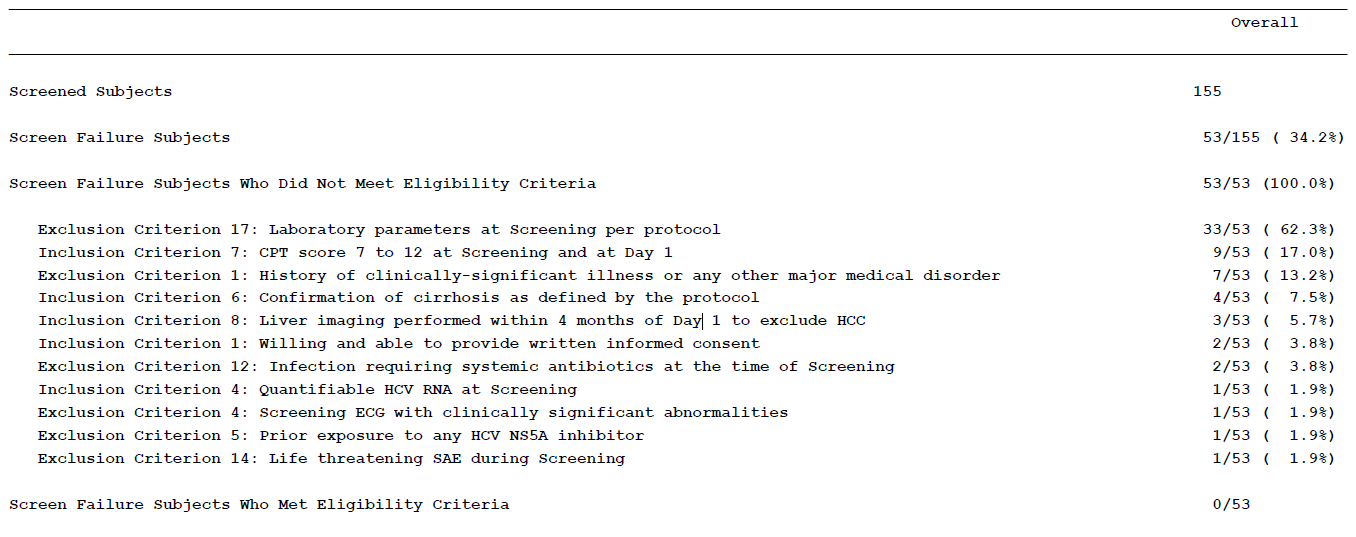


**Supplemental Table 3. Characteristics of patients with virologic relapse**

| **Study Treatment** | **Age** | **Sex** | **BMI (kg/m^2^)** | **Genotype** | **Baseline**  **CPT Class** | **IL28B** | **Baseline HCV RNA (log_10_ IU/mL)** | **Timing of VF (FU Week)** | **Prior HCV Treatment** | **NS5A Resistance-associated substitutions** | |
| --- | --- | --- | --- | --- | --- | --- | --- | --- | --- | --- | --- |
|  |  |  |  |  |  |  |  |  |  | **BL** | **FU** |
| **SOF/VEL 12 Weeks** | 73 | F | 28.9 | 3b | B | CC | 5.68 | 4 | IFN | A30K, L31M | A30K, L31M |
|  | 60 | M | 25.1 | 2b | C | CC | 6.17 | 4 | Peg-IFN, RBV | L31M | L31**I**/M, **Y93H** |
|  | 58 | F | 29.5 | 1a | C | CT | 5.90 | 4 | Peg-IFN, RBV | None | **Y93N** |
|  | 48 | M | 31.3 | 1b | B | TT | 6.00 | 12 | Treatment-Naïve | L31L/M | **L31I, Y93H** |
| **SOF/VEL+RBV 12 Weeks** | 75 | F | 24.2 | 1b | B | CC | 5.45 | 12 | Treatment-Naïve | L28M, Y93Y/N | L28M, Y93N |
|  | 51 | M | 27.4 | 1b | C | CT | 6.46 | 12 | IFN, Peg-IFN, RBV, simeprevir | None | **L31V, Y93H** |

Note: Bold font represents treatment-emergent RASs.

BMI, body mass index; CPT, Child-Pugh Turcotte; FU, follow-up; HCV, hepatitis C virus; IFN, interferon; RBV, ribavirin; SOF, sofosbuvir; VEL, velpatasvir;

**Supplemental Table 4. Components of CPT for Patients with Improvement in CPT Score**


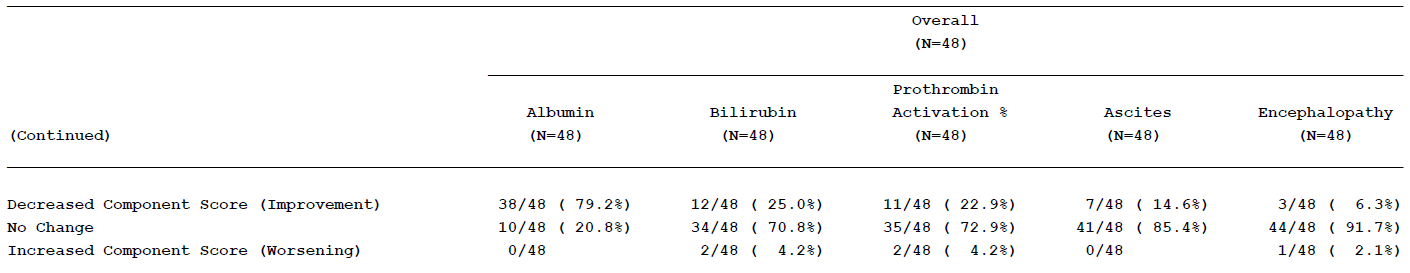


**Supplemental Table 5. Serious Adverse Events**


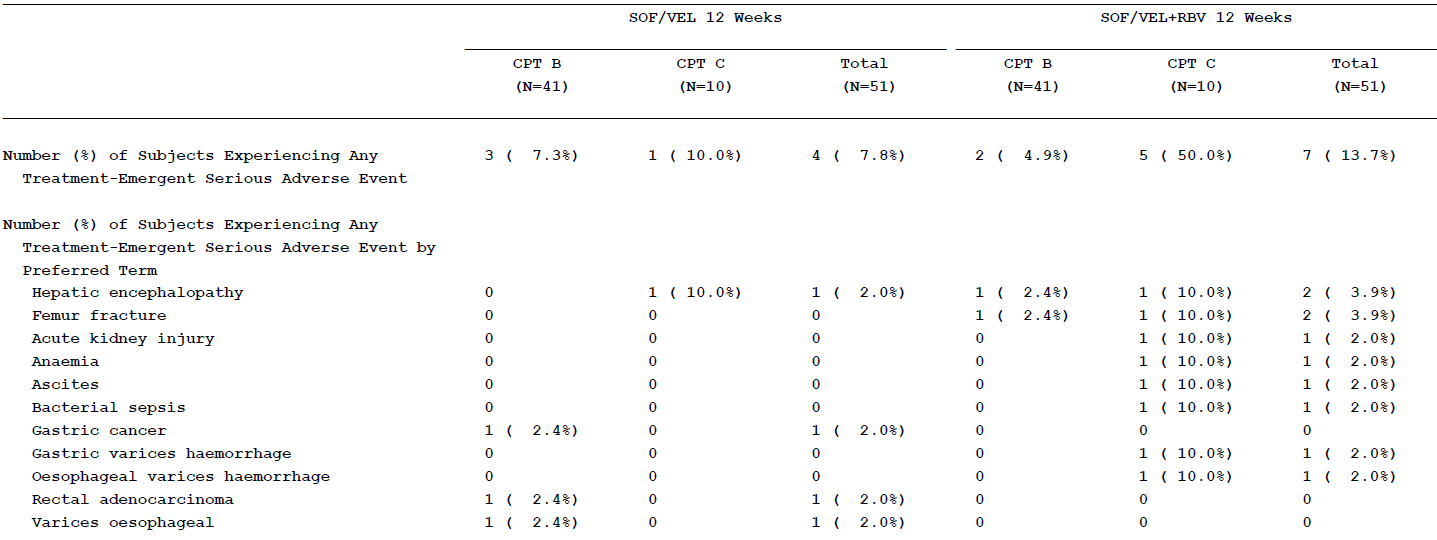


**Supplemental Table 6. Deaths**

| **Treatment group** | **Cause** | **Comments** |
| --- | --- | --- |
| SOF/VEL+RBV 12 Weeks | Septicemia gram-negative | The 59-year old female with CPT class C cirrhosis at baseline was hospitalized on Day 2 for esophageal varices hemorrhage (Grade 3 SAE) which led to ribavirin discontinuation on Day 2 and sofosbuvir-velpatasvir interruption from Day 2 to Day 8.On Day 43 during the same prolonged hospitalization, sofosbuvir-velpatasvir was discontinued due to acute kidney injury (Grade 2 SAE). On posttreatment Day 3, the subject experienced a Grade 2 AE bacterial sepsis which became a Grade 4 SAE on posttreatment Day 5, and was the cause of death on posttreatment Day 5. |
| SOF/VEL+RBV 12 Weeks | Hepatocellular carcinoma | The 51-year old male with CPT class C cirrhosis at baseline experienced a nontreatment-emergent Grade 3 SAE of HCC on posttreatment Day 70 which became a Grade 4 SAE on posttreatment Day 155 and was the cause of death on posttreatment Day 158. |
| SOF/VEL+RBV 12 Weeks | Gastric varices hemorrhage | The 67-year old male with a history of gastric varices and CPT class C cirrhosis at baseline experienced a Grade 3 AE of gastric varices hemorrhage on Day 50 which led to discontinuation of all study drugs on Day 50, became a Grade 4 SAE on posttreatment Day 1,and was the cause of death on posttreatment Day 17. |

**Supplemental Figure 2. Selected laboratory parameters during study treatment**

**Lymphocytes**


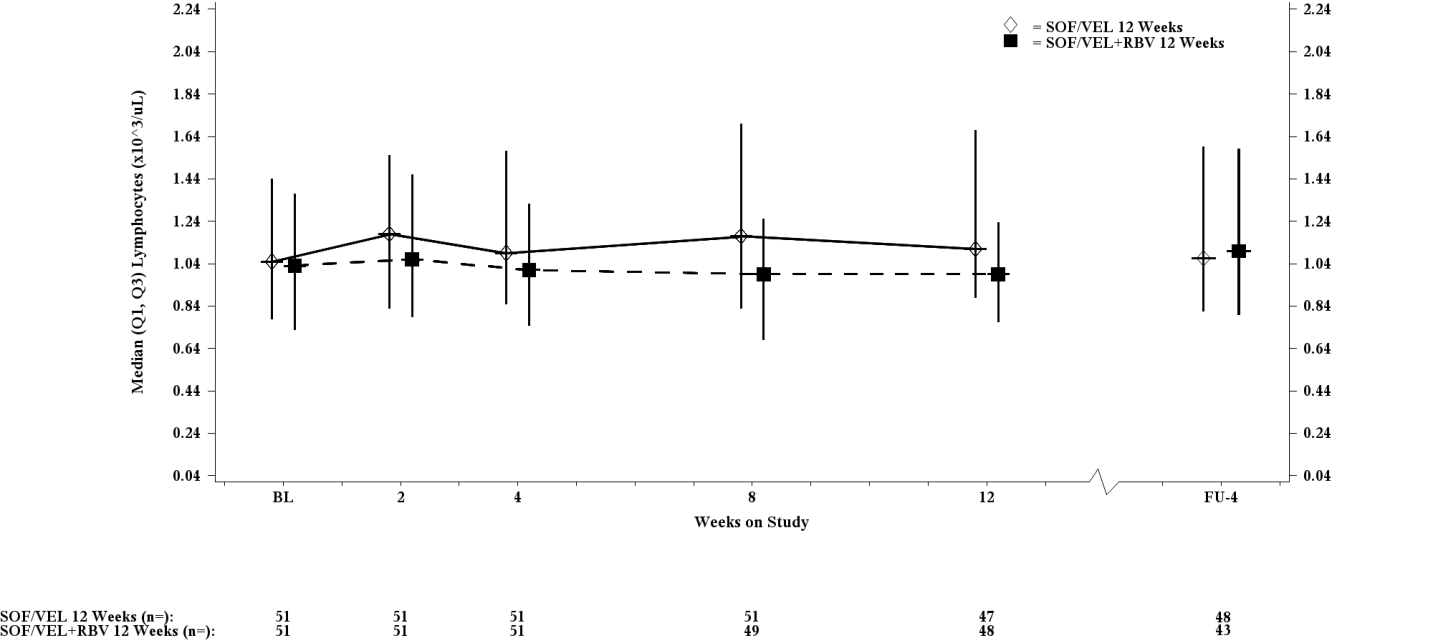


**Hemoglobin**


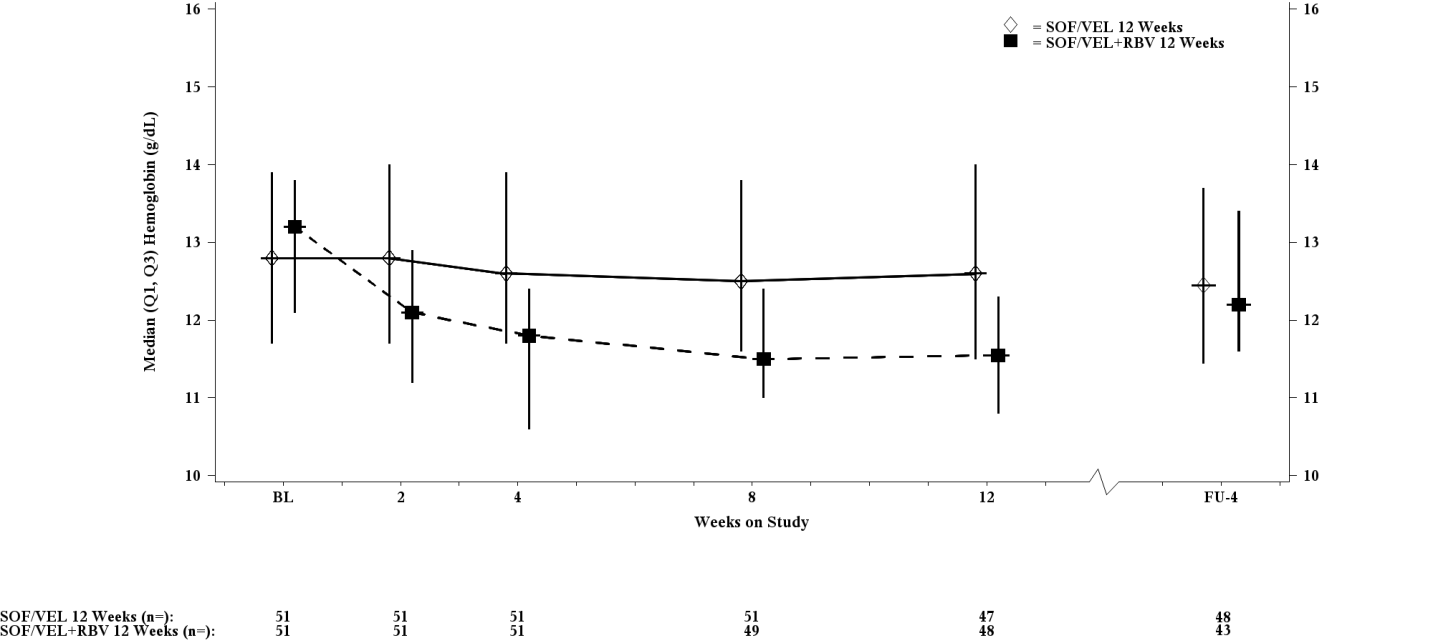


**Reticulocytes**


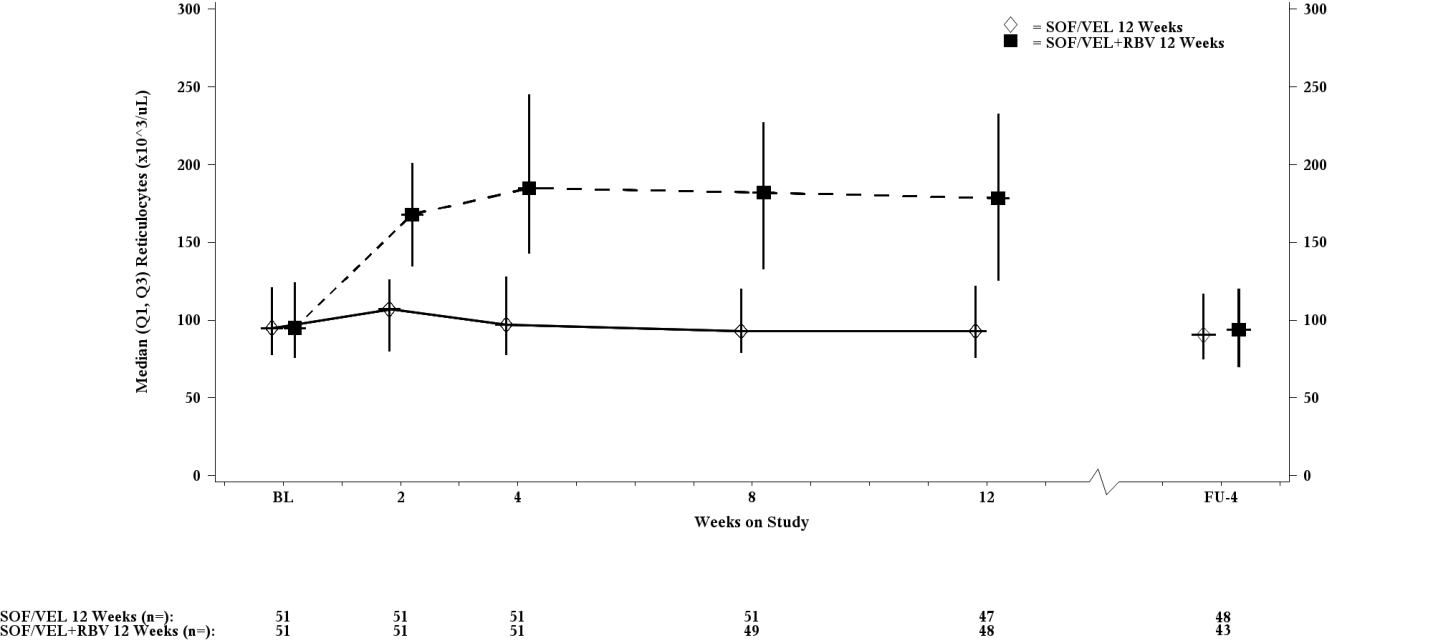


**Platelets**


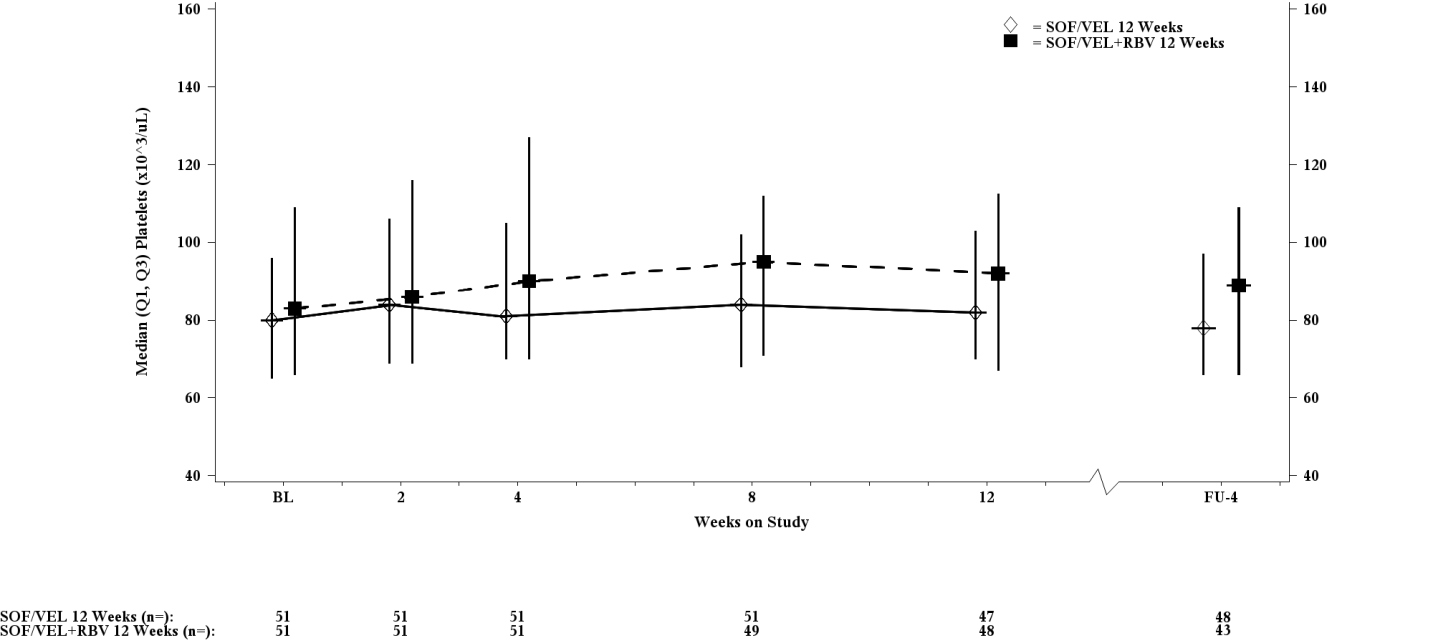


**Alanine Aminotransferase**
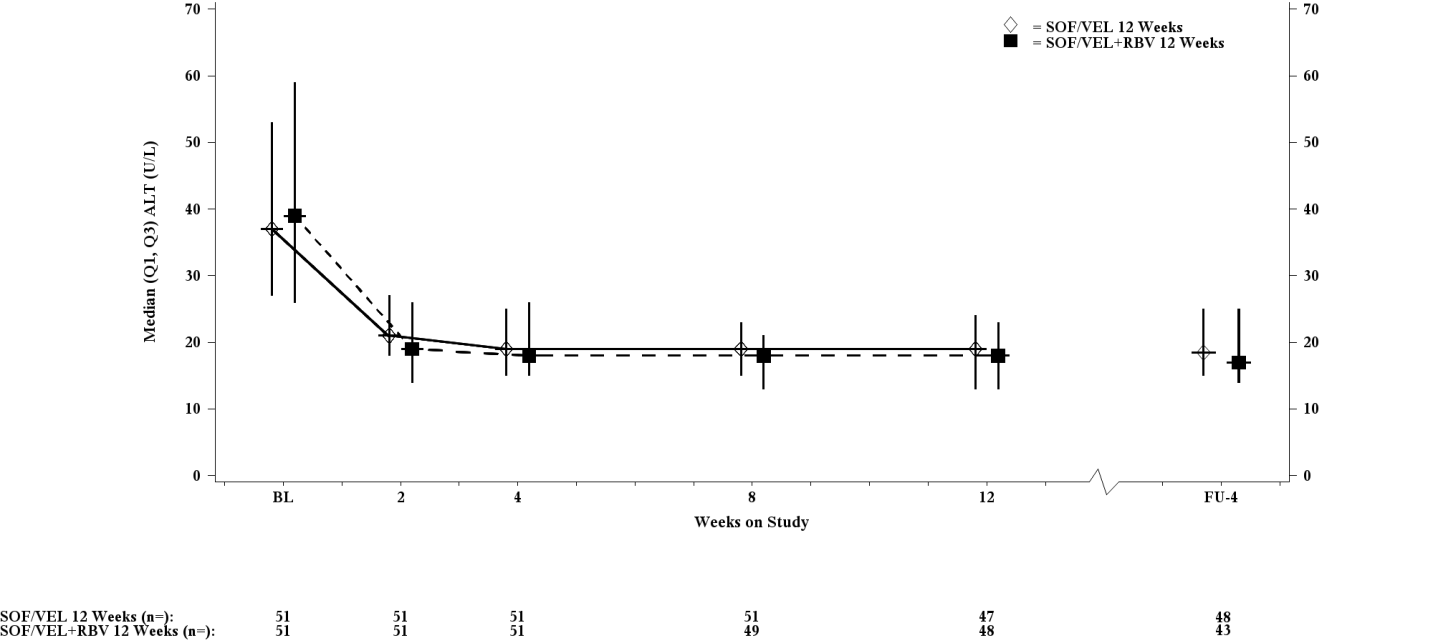


**Total Bilirubin**


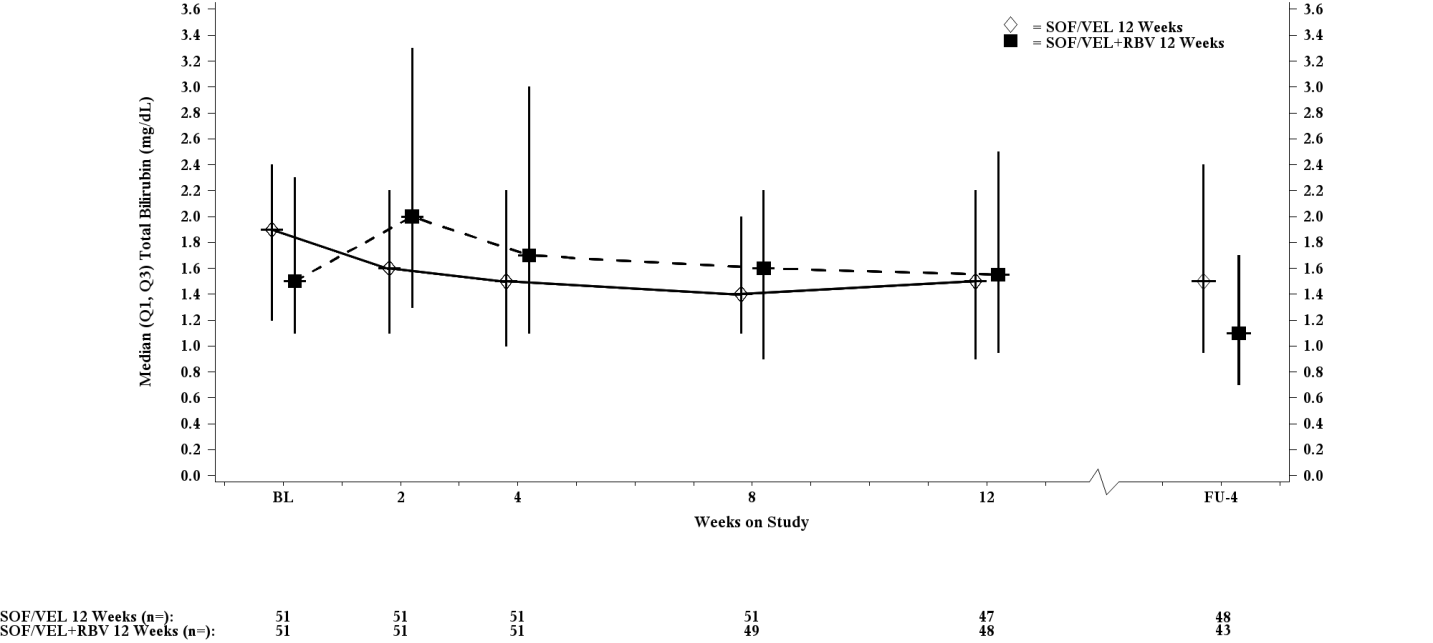

Supplement: Supplementary file 1 — Supplementary material 1 (DOCX 572 kb) [file 535_2018_1503_MOESM1_ESM.docx]
